# Grape Berry Flavonoid Responses to High Bunch Temperatures Post Véraison: Effect of Intensity and Duration of Exposure

**DOI:** 10.3390/molecules24234341

**Published:** 2019-11-27

**Authors:** Julia C. Gouot, Jason P. Smith, Bruno P. Holzapfel, Celia Barril

**Affiliations:** 1 National Wine and Grape Industry Centre, Wagga Wagga, NSW 2678, Australia; jpsmith@csu.edu.au (J.P.S.); bruno.holzapfel@dpi.nsw.gov.au (B.P.H.); cbarril@csu.edu.au (C.B.); 2 School of Agricultural and Wine Sciences, Charles Sturt University, Wagga Wagga, NSW 2678, Australia; 3 Charles Sturt University, Leeds Parade, Orange, NSW 2800, Australia; 4 New South Wales Department of Primary Industries, Wagga Wagga, NSW 2650, Australia

**Keywords:** anthocyanin, Doehlert design, high temperature, flavonoid, flavonol, flavan-3-ol, grape, tannin, threshold

## Abstract

Climate models predict an increase in the frequency and duration of heatwaves with an increase in intensity already strongly evident worldwide. The aim of this work was to evaluate the effect of two heatwave-related parameters (intensity and duration) during berry ripening and identify a threshold for berry survival and flavonoid accumulation. A Doehlert experimental design was used to test three temperature intensities (maxima of 35, 46, and 54 °C) and five durations (3 to 39 h), with treatments applied at the bunch level shortly after véraison. Berry skin and seeds were analysed by liquid chromatography-triple quadrupole-mass spectrometry (LC-QqQ-MS) for flavonoids (flavonols, anthocyanins, free flavan-3-ols, and tannins). Berries exposed to 46 °C showed little difference compared to 35 °C. However, berries reaching temperatures around 54 °C were completely desiccated, and all flavonoids were significantly decreased except for skin flavonols on a per berry basis and seed tannins in most cases. Some compounds, such as dihydroxylated flavonoids and galloylated flavan-3-ols (free and polymerised), were in higher proportion in damaged berries suggesting they were less degraded or more synthesised upon heating. Overall, irreversible berry damages and substantial compositional changes were observed and the berry survival threshold was estimated at around 50–53 °C for mid-ripe Shiraz berries, regardless of the duration of exposure.

## 1. Introduction

Heatwaves are extreme events; they can be occasional and sometimes unpredictable, and the impacts can be severe. Various definitions of heatwave are reported in the literature and can be collectively summarised as a period of unusually and excessively high temperatures compared to a given threshold [1]. Intensity (average and maximum temperatures) is the most important parameter to consider when studying heatwaves. The period of the year at which they occur, for example winter versus summer, can be used to refine threshold values [2]. Location is also important, as what constitutes a heatwave in one location might be considered normal elsewhere. Frequency is another parameter, and even though it may not be common, several heatwaves may occur within the same growing season. Lastly, duration is also critical as heatwaves can last from a day to several weeks. In the context of climate change, it is clear that increases in heatwave frequency, intensity, and duration have already taken place, and will continue to occur [3].

Under such conditions, agriculture, including viticulture, will be significantly impacted around the world, with heatwaves being a greater danger for wine grape production than an increase in average temperature as they can generate irreversible damage [4,5]. In addition to reduced yields, a decrease in grape quality and subsequent wine value is expected as temperature impacts berry metabolism, and reshapes the profile of major groups of compounds. In general, wine quality is balanced based on the technological maturity driven by sugars and organic acids, the phenolic maturity of compounds contributing to colour and mouthfeel, and the aromatic profile which depends on a wide range of volatile and non-volatile compounds. Fermentation processes can also be influenced by the amount and type of nitrogenous compounds such as amino acids present in grapes. Hence, numerous studies have been conducted to understand the impact of high temperature on grape composition. Changes reported include increased sugars, degradation of organic acids, a decrease in polyphenols, and modification of volatile and amino acid profiles [6,7,8].

In particular, flavonoids, a polyphenol sub-family, have attracted significant research attention in recent decades. Anthocyanins, the red pigments in the skin of red varieties, are now well known to be reduced under high temperature. This occurs either directly or indirectly due to altered carbon allocation, lower synthesis, and/or enhanced degradation [9]. However, variable results have been reported for flavonols, which are also present in the skin, and primarily act as UV-protectors. Free flavan-3-ols and tannins (polymers of flavan-3-ols) in skin and seeds have been either inconsistently affected, or not measured in various studies, suggesting an opportunity for further research attention into the temperature responses of these compounds. While temperature effects on grape composition have been extensively studied, the effect of the combinations of several levels of high temperature intensity and duration of exposure are also yet to be tested experimentally.

The aim of this work was therefore to identify a temperature threshold for berry survival and flavonoid accumulation, i.e., the temperature at which berry damage become irreversible, and/or at which secondary metabolites are impacted. In addition, the work aimed to assess the effect of high temperature on detailed tannin composition during the maturation phase and to evaluate the extent of the impact of different intensities and durations on all flavonoids using a Doehlert design. Such an experimental design is more commonly used in chemistry and biotechnology, allowing a more efficient optimisation procedure involving multiple experimental variables [10,11]. In this study, bunch temperature was manipulated using an active heating system generating different temperature differentials (+8.4, +16.7 °C) for a duration varying between 3 and 39 h and detailed tannin composition, after polymer cleavage in skin and seeds, as well as many flavonoids in the skin were measured.

At maturity, berries that remained under 50 °C were intact and flavonoid composition was unchanged, however all berries that reached temperature above 53 °C, regardless of the duration, were desiccated with dramatic reduction of flavonoids and changes in profile. The 50–53 °C was a window of transition were berries were either damaged or not, and could be the threshold window for necrosis of mid-ripe Shiraz berries.

## 2. Results and Discussion

### 2.1. Experimental Design and Temperature Treatments

For the first time in a grapevine physiology context, a complex experimental design, the Doehlert design, was chosen to study the effect of several combinations of temperature-related parameters: duration and intensity of exposure [10,11]. The Doehlert design, also known as a uniform shell design, was selected due to its flexibility and allowance for experimental values (recorded during the experiment) to slightly differ from targeted values (calculated with the design) (Table 1).

Days after the onset of véraison (DAV+) were used to indicate treatment and berry sampling periods. Treatments were applied after the onset of véraison (DAV+15–17) which has been previously identified as a sensitive window for flavonoids as a multitude of genes involved in anthocyanin and flavonol biosynthesis are activated [7,9]. The temperature difference (dT) was used to describe the seven treatments: three temperature intensities (ambient, AT; high, HT; very high temperature, VHT) combined with five durations (3–39 h) (Table 1). As explained in Gouot, et al. [12], the heating system can successfully manipulate berry surface temperature, and in this experiment values measured by thermal imaging were close to the temperature of the air blown onto the bunches (y = 0.9184x + 1.6531, R^2^ = 0.9212). Hence, estimation of bunch temperature with the thermocouples was used to characterise the treatments as thermal images were only taken at key timings. AT treatment temperatures averaged 31.2 and 31.6 °C for 12 and 30 h, respectively, and both treatments remained under 36 °C during the 3-day experiment period. As dT was calculated from the difference of the treated bunch compared to an untreated bunch on each vine, experimental values could slightly drift from the background temperature as they depended on the position of each bunch with dT_AT/12h_ = +0.05 and dT_AT/30h_ = −1.8 °C. HT treatments were set to a target of +8.4 °C, although actual temperatures spanned +7.3–10.2 °C between the three durations tested (3, 21, and 39 h). The average temperature was calculated over the heating period, and hence averaged 43.8 °C for HT/3h (applied at the hottest time of day) while HT/21h and HT/39h only averaged 37.6 and 39.7 °C, respectively. Very hot air was blown onto the bunches for the +16.7 °C for VHT, with VHT/12h reaching a maximum of +19.8 °C whereas VHT/30h only reached +14.6 °C. Average temperatures for both treatments also slightly differed with 49.9 °C versus 46.6 °C, respectively. Maximum temperatures for each HT and each VHT treatment were close to each other regardless of the duration of exposure as the second day was the warmest and the highest maximum recorded for HT, 3, 21, and 39 h was 45.7, 45.1, and 46.7 °C, respectively, while VHT reached 52.2 (30 h) and 55.1 °C (12 h).

**Figure 1:**
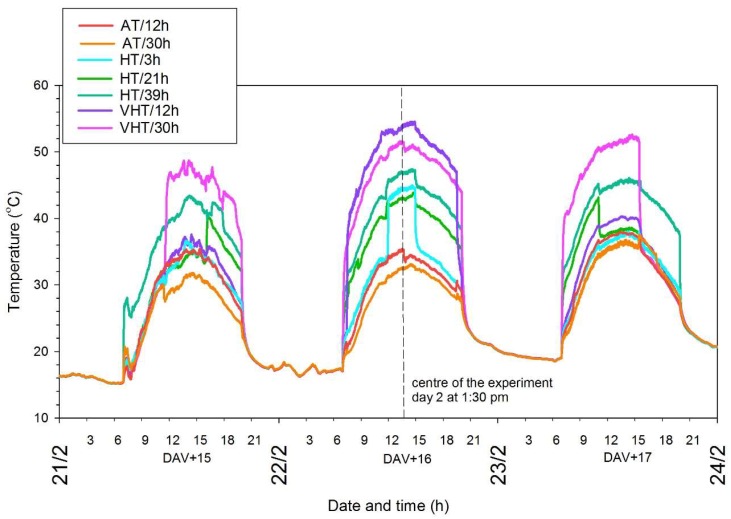
Air temperature records (5 s intervals) during the three days of the experiment averaged per treatment (*n* = 4, except for VHT/12h (*n* = 3) and HT/21h (*n* = 9)) which combined three intensities (AT: ambient temperature; HT: high temperature; VHT: very high temperature) and five durations of exposure (varying between 3 and 39 h). The period of treatment application is indicated with the number of days after the onset of véraison (DAV+15–17).

Bunches exposed to heating over a full day (12 h) or full three days (39 h) had their temperature increased slowly in the morning (Figure 1). For the shortest heating period, there would have been 37 timing possibilities applying 3 h of heating over a 39 h-period, so it was decided all durations would be centred on day 2 (dotted line, Figure 1). Thus, for the shortest duration (3h) which started at 12 p.m. on day 2, the temperature quickly jumped from 34 to 42 °C and exposures of 30 and 21 h, which started at 11:30 a.m. and 4 p.m. on day 1, respectively, also exhibited rapid increases in temperature. The possibility of rising the temperature slowly by gradual ramping to avoid jumps of temperature has been previously mentioned [13]. However, in a vineyard, relatively rapid changes in temperature could also occur with conditions of changing cloud cover during heatwaves or if bunch exposure differs during the course of the day with row orientation [4,14]. The AT treatments matched weather conditions around véraison in Australia, while HT simulated a heatwave of high intensity, and VHT replicated an extreme heatwave with a maximum above 50 °C. Such air temperatures have not yet been recorded in grape growing regions, but the skin of dark-coloured grape berries can reach such temperatures if exposed to full sunlight [15,16]. Hence, the temperature parameters realistically mimicked heatwaves happening around véraison, i.e., December-January, in warm and hot Australian grape growing regions [17].

By testing three different temperature intensities, including one that was excessively high, the aim was to identify a maximum temperature and minimum duration threshold for berry survival. Due to the small variations in temperature between biological replicates, individual berry weight for all samples at harvest (including the two treatment replicates that “failed”, see 4.3.4) could be plotted against maximum temperature, highlighting that damage started to appear and be irreversible in the 50–53 °C temperature window (data not shown). This type of damage, also called sunburn necrosis, has been previously observed on apples exposed to extreme high temperature (from 52 °C) and was also generated in the absence of excessive light [18], similar to the present experiment where light and UV were close to outside conditions.

### 2.2. Berry Physiology Responses to Different Temperature Intensities and Durations

**Figure 2:**
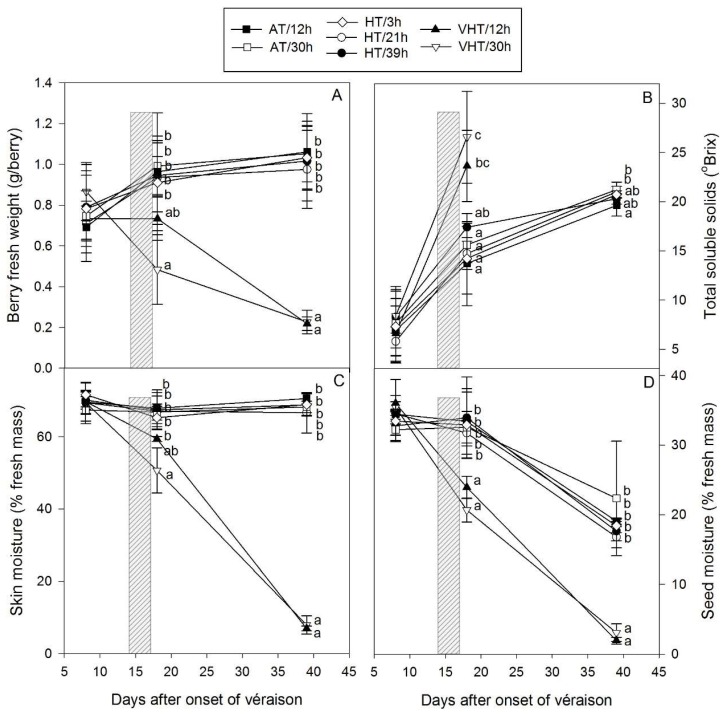
Effect of treatments (temperature intensity: AT, ambient temperature; HT, high temperature; VHT, very high temperature and duration of exposure: varying between 3 and 39 h) on berry fresh weight (**A**), total soluble solids (**B**), skin % moisture (**C**), and seed % moisture (**D**) average (mean ± SD, *n* = 4, except for VHT/12h (*n* = 3) and HT/21h (*n* = 9)). The period of heating treatment (15–17 days after the onset of véraison) is indicated with a grey rectangle. Significant differences between treatments were tested by analysis of variance followed by Tukey test (honestly significant difference) for each sampling date and are indicated with lower case letters (*p* < 0.05). Total soluble solids could not be measured for VHT treatments at maturity as berries were too desiccated.

Prior to treatment application after the onset of véraison, berries averaged 0.8 g and 7 °Brix at DAV+8 and, under AT and HT, kept enlarging to reach about 1 g at maturity (DAV+39) with total soluble solids (TSS) spanning 19.7–21.2 °Brix (Figure 2A,B). All AT and HT berry growth parameters exhibited a similar pattern regardless of the intensity and the duration of exposure and did not show any differences between treatments. In contrast, berries exposed to VHT desiccated rapidly following the treatments. At DAV+18, 12 h of VHT led to lower berry weight (0.73 g), while berries exposed for 30 h were already visibly shrivelled and berry weight was reduced to 0.48 g. At DAV+39, berry weights had declined to only 0.22 g for both durations (Figure 2A), and consisted of just dry skin and seeds. Similar damage has been observed in the field after the severe 2009 Australian summer heatwave where air temperatures up to 48.2 °C were recorded in grape production regions [4]. In our study, TSS increased quickly during treatment application from 7.2 °Brix at DAV+8 to about 25 °Brix at DAV+18 for VHT, while TSS for AT and HT berries increased more slowly to 15.1 °Brix. The reduction in berry moisture and subsequent juice in VHT was driving this earlier TSS increase as the amount of sugar per berry was the same across all treatments (data not shown). By the time the other treatments reached maturity, VHT berries had completely desiccated and TSS could not be measured (Figure 2B). On the other hand, small differences were found between AT and HT berries with HT/21h and AT/30h significantly higher than AT/12h (Figure 2B). 

The percentage of moisture in skin and seeds (Figure 2C,D) was unaffected by AT and HT with skin moisture constant across berry ripening (65%–72%) and seed moisture decreasing slowly from 35% (DAV+8) to 19% (DAV+39) due to the typical seed maturation process including browning and loss of water [19]. In agreement with the reduction in berry weight and desiccation due to VHT exposure, loss of skin and seed moisture was substantial for those treatments, reaching 7.5 and 2.5% by DAV+39, respectively. Browning of the bunch stem, pedicel and skin of the berries appeared within a few hours of heating, as previously observed [12]. Thus, VHT/30h damage appeared on day 1 and because the treatment started at 11 a.m., berries were rapidly exposed to the daily maximum which usually occurs between 2 and 3 p.m. In addition, with damage to the pedicel and rachis potentially limiting the import of water into the berries, the extreme high vapour pressure deficit recorded on day 2 and 3 (Supplementary Materials, Table S1) would have partially contributed to the evaporation of the berry water. In contrast, VHT/12h heating treatment only started on day 2. While visual heat damage symptoms also appeared quickly, they were not exacerbated by another day of heating and berries would have been expected to shrivel more slowly. However, at DAV+18, berry weights and percentages of moisture between VHT treatments were not statistically different, suggesting that VHT/12h berries shrivelled as much as VHT/30h. Indeed, the difference in temperature (dT, mean and max) between VHT/12h and VHT/30h could have contributed to this phenomenon.

As opposed to VHT, HT berries were not visibly damaged. This would be consistent with previous observations that post-véraison grapes seem to be less sensitive to heat damage than those going through véraison [4]. In the current study, berries were already quite advanced (10 °Brix) as the treatment was imposed 2 weeks after the onset of véraison. With a high but not unprecedented maximum of 46 °C for HT berries, this finding is also in agreement with general industry experience that fruit can tolerate such temperatures providing vines are well irrigated [4]. 

### 2.3. Temperature Effect on Total Skin Flavonoids

**Figure 3:**
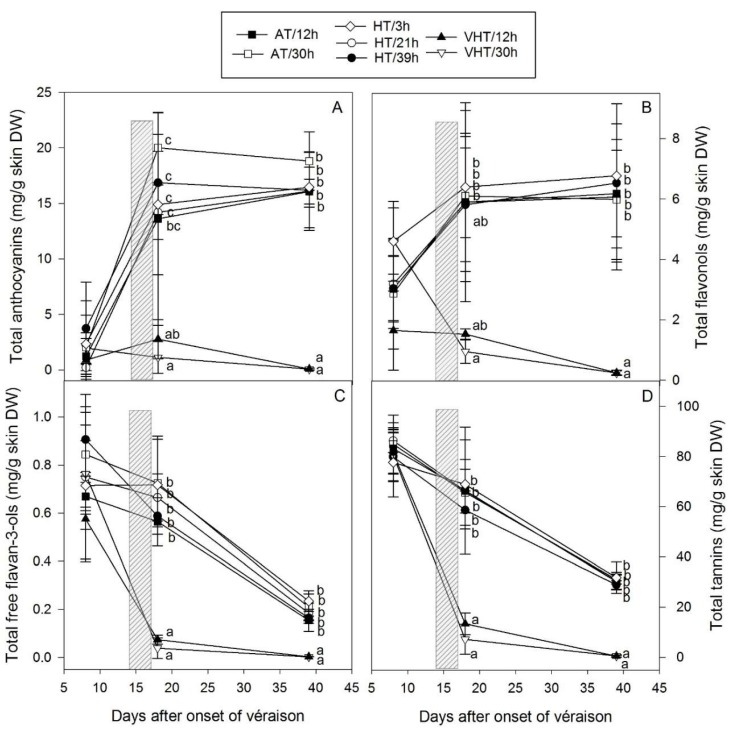
Effect of treatments (temperature intensity: AT, ambient temperature; HT, high temperature; VHT, very high temperature and duration of exposure: varying between 3 and 39 h) on total skin anthocyanins (**A**), flavonols (**B**), flavan-3-ols (**C**), and tannins (**D**) expressed on dry weight (DW) basis (mean ± SD, *n* = 4, except for VHT/12h (*n* = 3) and HT/21h (*n* = 9)). The period of heating treatment (15–17 days after the onset of véraison) is indicated with a grey rectangle. Significant differences between treatments were tested by analysis of variance followed by Tukey test (honestly significant difference) for each sampling date and are indicated with lower case letters (*p* < 0.05).

Different sub-families of flavonoids were measured in the skin (see supporting data—Table S2) with the total concentrations of the main four, native anthocyanins, flavonols, free flavanols, and tannins, calculated by addition of all individual compounds on a dry weight (DW) basis (Figure 3). Variations in concentrations were observed between treatments at DAV+8, due to heterogeneity before berry thinning, however these were not significant. Anthocyanins, the main red pigments found in grapes, were in low concentration in berries that just started to turn red (ranged 0.2–3.7 mg/g) but increased rapidly during berry ripening to about 17.1 mg/g skin under AT and HT (Figure 3A). Flavonols were also found in low concentrations before treatment application, with values spanning 1.7–4.6 mg/g skin at DAV+8, and increasing slowly during ripening to about 6.2 mg/g skin at DAV+18 and DAV+39 under AT and HT (Figure 3B). At DAV+8, while free flavan-3-ols were only found in small amount (less than 1 mg/g) in the skin (Figure 3C), tannins were by far the most abundant flavonoids (Figure 3D). For AT and HT treatments, flavan-3-ols and tannins ranged 0.60–0.95 and 80 mg/g skin at DAV+8 and both decreased slowly to 0.2 and 35 mg/g at DAV+39, respectively (Figure 3C,D). 

In general, the total concentration of all sub-families was decreased after VHT with 12 h of exposure being as critical as 30 h. Under VHT, anthocyanins and flavonols did not accumulate or were degraded immediately (Figure 3A,B), and the decrease of flavan-3-ols and tannins was substantially accelerated (Figure 3C,D). At DAV+39, all flavonoid concentrations were below 1 mg/g skin with no differences between 12 and 30 h of exposure while at DAV+18, under VHT/12h all concentrations were slightly higher, although not significantly. 

**Figure A1:**
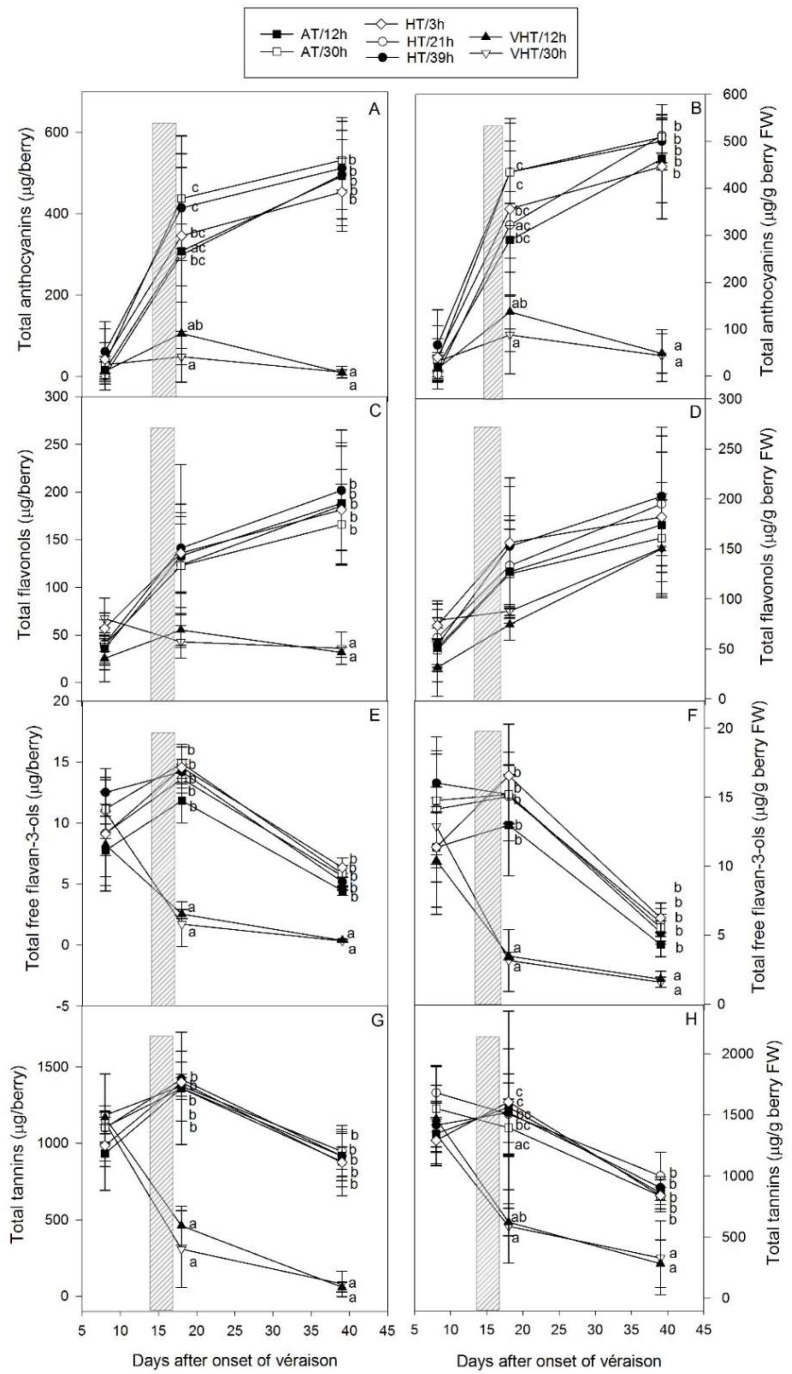
Effect of treatments (temperature intensity: AT, ambient temperature; HT, high temperature; VHT, very high temperature and duration of exposure: varying between 3 and 39 h) on total anthocyanins (mean ± SD, *n* = 4, except for VHT/12h (*n* = 3) and HT/21h (*n* = 9)) expressed per berry (**A**) and on a berry fresh weight (FW) basis (**B**), total flavonols per berry (**C**) and on a berry FW basis (**D**), total flavan-3-ols per berry (**E**) and on a berry FW basis (**F**), and total tannins per berry (**G**) and on a berry FW basis (**H**). The period of heating treatment (15–17 days after the onset of véraison) is indicated with a grey rectangle. Significant differences between treatments were tested by analysis of variance followed by Tukey test (honestly significant difference) for each sampling date and are indicated with lower case letters (*p* < 0.05).

The patterns of accumulation and differences in flavonoids induced by VHT on a DW basis were similarly observed on a content and fresh weight (FW) bases (Figure A1). While the anthocyanin content was reduced 50-fold and free flavan-3-ol and tannin contents were decreased about 15-fold under VHT at DAV+39, flavonols were also lower, but only 5-fold (Figure A1—left hand side). Interestingly, when concentrations were expressed per gram berry FW (Figure A1—right hand side), anthocyanins, flavan-3-ols and tannins remained significantly lower in VHT-treated berries at DAV+39 while there were no differences in flavonols as VHT berries were about five times smaller. This could confirm the hypothesis that flavonols are still affected by temperature but to a lower extent than other flavonoids as suggested by the literature [9,20,21] and highlights the importance of expressing content and concentration values in different units. Anthocyanins were affected by VHT, however, surprisingly, HT, with temperature reaching 46 °C for three days, did not impact Shiraz in this study. This is in contrast with previous observations for Merlot bunches exposed to temperature above 40 °C (maximum of 43.3 °C), although in this case, treatments started one to three weeks prior véraison until maturity, and hence included the period of maximum anthocyanin production [20]. In the present experiment, treatments may have been applied just after the most sensitive period for anthocyanin biosynthesis explaining the contrast to anthocyanin responses reported in the literature [9]. Finally, tannins and free flavan-3-ols were clearly impacted by extreme heat but not under HT.

The aim of using a Doehlert design was to predict the least favourable conditions for flavonoid accumulation, by evaluating the extent of the impact of two heatwave-related parameters (intensity and duration) on flavonoid concentrations. Multi linear regressions (MLRs) performed using the design parameters showed the importance of temperature over the duration of exposure (Table 2). At DAV+18, coefficients for constant and dT terms were significant for all flavonoids and a significant interaction between dT and duration was also found for anthocyanins, whereas at DAV+39, only the coefficients for constant and temperature were significant. In general, the models created using the coded Doehlert parameters did not well predict total flavonoids with R^2^ values ranging from 0.193 to 0.597. The best regressions were found for anthocyanins, flavan-3-ols and tannins at maturity, and the worst for flavonols at both sampling dates. This tends to confirm the direct effect of temperature on anthocyanins, flavan-3-ols, and tannins, as opposed to flavonols, which were most likely indirectly degraded following berry death. 

In order to identify a temperature threshold for berry flavonoid accumulation, simple regressions using maximum temperatures were also tested for all flavonoid sub-families. They only showed good prediction for total anthocyanins and tannins at harvest and the best regressions were polynomials (data not shown) with R^2^ = 0.798 and R^2^ = 0.806, respectively. Interestingly, these simpler models provided a better prediction of flavonoid composition than MLR as highlighted by the higher R^2^ values (Table 2). As observed for berry weight, the maximum temperature reached by the berries seemed to have been the most critical as flavonoid composition started to be substantially altered in the same 50–53 °C temperature window (see Section 2.1). 

### 2.4. Temperature Effect on Skin Flavonoid Composition

**Figure 4:**
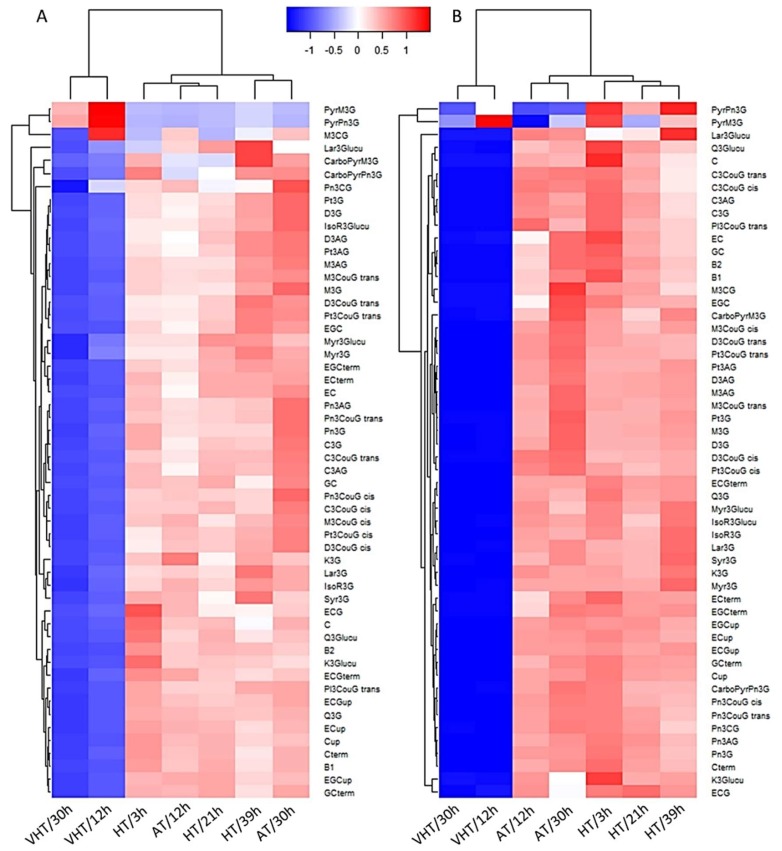
Heatmap of grape skin flavonoids including tannin subunits just after treatment application (**A**) and at maturity (**B**) with several temperature intensity treatments: ambient (AT), high (HT) and very high temperature (VHT); and several durations of exposure (from 3 to 39 h). The heatmap was generated using the normalised mean values of four biological replicates (except *n*_VHT/12h_ = 3 and *n*_HT/21h_ = 9), with in blue, concentrations below, and in red concentrations above averages across treatments. Abbreviations: *Anthocyanins (A)*. C, Cyanidin; D, Delphinidin; M, Malvidin; Pn, Peonidin; Pt, Petunidin; A3G, A-3-*O*-glucoside; A3AG, A-3-*O*-acetyl-glucoside; A3couG, A-3-*O*-(6″-*p*-coumaroyl-glucoside); PyrA3G; PyranoA-3-*O*-glucoside; CarboPyrA3G; CarboxypyranoA-3-*O*-glucoside; *Flavonols (F)*. Myr, Myricetin; Q, Quercetin; K, Kaempferol; Syr, Syringetin; IsoR, Isorhamnetin; Lar, Laricitin; F3Glucu, F-3-*O*-glucuronide; Flavan-3-ols. C, (+)-Catechin; EC, (−)-Epicatechin; ECG, (−)-Epicatechin gallate; GC, (+)-Gallocatechin; EGC, (−)-Epigallocatechin; term, terminal subunit; up, upper subunits; B1/2, Procyanidin B1/2.

Most compounds in the skin on a DW basis, including individual native anthocyanins, flavonols, free flavan-3-ols, and those involved in tannin polymerisation were significantly decreased by VHT at DAV+18, immediately after treatment application, with VHT/30h generally more affected than VHT/12h, as represented by the deeper blue colour (Figure 4A). Pyranoanthocyanins were an exception with an increase of pyranomalvidin-3-*O*-glucoside and pyranopeonidin-3-*O*-glucoside (B-type vitisins) under VHT (and of a higher magnitude under VHT/12h) but no increase in carboxypyranoanthocyanins (A-type vitisins). Malvidin-3-*O*-acetylglucoside was also increased under VHT/12h but decreased under VHT/30h. HT treatments were clustered together with AT with most compounds close to or above average. AT/30h exhibited the highest concentration of compounds from peonidin-3-*O*-acetylglucoside to delphinidin-3-*O*-coumaroylglucsoide as grouped in Figure 4 while the cluster (−)-epicatechin gallate (free) to (+)-gallocatechin (terminal subunit) were the most abundant in HT/3h.

At maturity (DAV+39), treatments were clustered into three distinct groups based on temperature intensity (Figure 4B). AT treatments were grouped together with AT/30h exhibiting the highest concentrations of trihydroxylated anthocyanins (delphinidins, petunidins, and malvidins). This treatment may have enhanced anthocyanin biosynthesis or higher concentrations were an artefact as AT/30h was also exhibiting the highest TSS overall (Figure 2B). However, HT/21h, which had a very similar TSS, did not exhibit as high anthocyanin concentrations. VHT/12h and VHT/30h were separated from the other treatments with all compounds substantially reduced, apart from B-type vitisins for VHT/12h. However, there were no longer differences in the depth of blue observed for the two VHT treatments as all compounds were significantly decreased and concentrations were close to zero. B-type vitisins were also increased in HT treatments, except for pyranomalvidin-3-*O*-glucoside under HT/21h. Pyranoanthocyanins were first discovered in aged and port wines and then, occasionally found in grapes, though the latter is thought to be an artefact from incorrect storage or processing, with for example, methylpyranoanthocyanins formed upon acetone extraction [22,23,24]. They have now been detected in several fresh and frozen grape samples and their formation suggested to occur in vivo with vitisins deriving from anthocyanins and acetaldehyde or pyruvic acid present in the cells of the berries [25,26,27]. Hence, in the present experiment, high temperature could have triggered/enhanced their formations as suggested by the higher B-type vitisin concentrations under VHT immediately after treatment application. The same reactions could have also occurred in HT berries but more slowly as B-type vitisins were also found in greater concentrations, but only at maturity.

Compounds were mainly clustered into two groups, those in the first two or three rows (depending on the sampling date) and the others (Figure 4A,B). In the second cluster, further groupings were identified based on high abundance. At DAV+18 (Figure 4A), clusters were found in AT/30h (in order, from petunidin-3-*O*-glucoside to delphinidin-3-*O*-coumaroylglucoside) and in HT/3h (in order from epicatechin gallate to gallocatechin terminal subunit). At DAV+39 (Figure 4B), flavan-3-ols and flavonols were mostly clustered together while anthocyanins were separated depending on their forms. For example, cyanidins were all grouped together as well as peonidins. These highlights the importance of the chemical properties of each sub-family as well as their respective branches along the phenylpropanoid pathway regarding their behaviour in grapes. Similar observations were made, for malvidins for example, in grapes under different sun exposure [15] or detached berries exposed to light or heat stress [28].

### 2.5. Temperature Effect on Skin Flavonoid Profile

**Figure 5:**
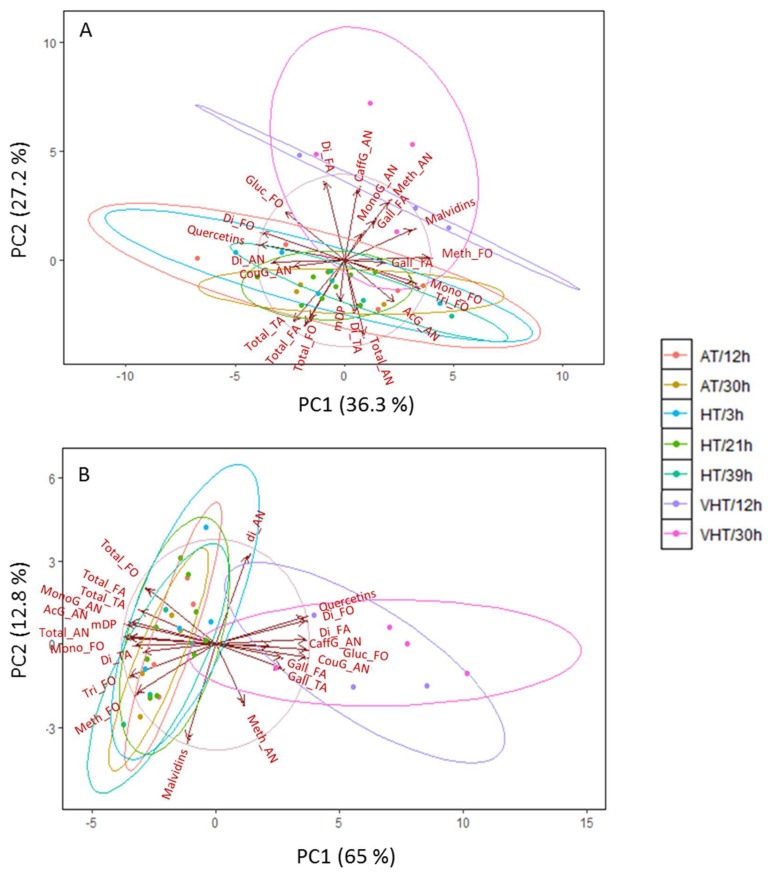
Principal component analysis (PCA) showing the impact of treatments (temperature intensity: AT, ambient temperature; HT, high temperature; VHT, very high temperature and duration of exposure: varying between 3 and 39 h) on the proportion of berry skin flavonoids just after treatment application (**A**) and at maturity (**B**) with dihydroxylated (Di_AN), monoglucoside (MonoG_AN), acetylglucoside (AcG_AN), coumaroylglucoside (CouG_AN), caffeoylglucoside (CaffG_AN), methylated (Meth_AN) and total (Total_AN) anthocyanins and malvidin proportions (Malvidins), mono- (Mono_FO), di- (Di_FO) and trihydroxylated (Tri_FO), methylated (Meth_FO), glucoside (Gluc_FO) and total (Total_FO) flavonols and quercetin proportions (Quercetins), galloylated (Gall_FA/Gall_TA), dihydroxylated (Di_FA/Di_TA) and total (Total_FA/TA) flavan-3-ols/tannins and mean degree of polymerisation (mDP).

Variables such as percentages of mono-, di-, and tri-hydroxylated (-OH on B ring) flavonoids and methylated (-CH_3_ on B ring) anthocyanins and flavonols, percentages of galloylation of flavan-3-ols and tannins and mean degree of polymerisation (mDP) were calculated as described in Pinasseau et al. [25]. In addition, other proportions specific to each group of flavonoids were determined: percentages of malvidins and quercetins or sub-families of anthocyanins: monoglucoside versus acylated with caffeoyl-, coumaroyl-, and acetylglucosides. Two principal component analyses (PCA) were conducted on the proportions of each flavonoid, per sampling date (Figure 5).

At DAV+18, only 63.5% of variation was represented by the biplot PC1-2 (Figure 5A). Immediately after treatment application, VHT treatments were already separated from the other treatments with VHT/12h the most clearly segregated. The separation along PC2 was concomitant with an increase in percentages of mono- and caffeoylglucosides as well as methylated anthocyanins and malvidins. Percentages of dihydroxylated free flavan-3-ols were also increased under VHT and negatively correlated with the percentage of dihydroxylated tannins. VHT treatments were separated from other treatment due to a higher proportion of flavan-3-ol galloylation and lower tannin mDP. In complement, a higher percentage of monoglucoside anthocyanins was also separating VHT/12h from VHT/30h along PC3, 8% (data not shown).

At maturity, 77.8% of variation was explained by PC1 and 2 (Figure 5B). The separation of VHT treatments was clear along PC1 and associated with firstly, a change in anthocyanin profile compared to other treatments with a decrease in acetylglucoside and monohydroxylated forms but an increase in proportion of coumaroyl forms. Effects on the malvidin family and methylated forms were no longer observed and malvidins were logically negatively correlated with dihydroxylated anthocyanins. Proportions of malvidins and dihydroxylated anthocyanins contributed the most to separation along PC2 and spread samples within treatments (AT and HT) suggesting variations of these compounds between biological replicates. Secondly, VHT had in general lower proportions of methylated, mono- and trihydroxylated flavonols concomitant with an increase in dihydroxylated and glucoside flavonols, and quercetin proportions. Lastly, both percentages of galloylation (free flavan-3-ols and tannins) were increased with VHT but mDP was reduced. Interestingly, dihydroxylated flavan-3-ols were again negatively correlated with dihydroxylated tannins, the latter decreased under VHT. 

In VHT treatments, bleaching of green berries (chlorophyll degradation) and browning of red berries on the first day suggested that reactive oxygen species (ROS) were rapidly produced [29]. Brown pigments, called oxidised polymeric phenolics, were possibly accumulated as a result but were not measured in this study. While some studies have concluded that flavonols are largely unaffected by temperature compared to light [20,30,31], an overview of the literature suggested they were sometimes directly [28] or indirectly impacted [9]. Flavonols can act as free-radical scavengers [29,32] and could react with ROS produced under extreme heat stress and thus still be degraded [33]. In addition, flavonols are synthesised at flowering but also after the onset of véraison [34] and throughout berry ripening. As flavonols have upstream genes in common with other flavonoids, their biosynthesis could also be indirectly affected. Slight changes in flavonol composition with higher proportion of dihydroxylated forms have been reported when Merlot grapes were cooled [35]. A lower proportion of myricetin but higher proportion of quercetin was found when Sangiovese vines were kept at low day temperature from véraison onwards [36]. In our study, flavonols were the most abundant flavonoids remaining in VHT grapes and their proportions were the most affected at maturity with predominantly, higher proportions of glucoside as opposed to glucuronide forms and quercetins inducing higher proportion of dihydroxylated forms. These results contrast with previous findings where whole vines and/or berries kept at lower temperature exhibited higher proportions of quercetin/dihydroxylated flavonols. However, in our study, VHT berries were completely desiccated and this could explain why flavonols behaved differently.

Anthocyanins are favoured by cooler temperatures [37,38] but are still produced under warmer conditions with some modulations [9]. The proportion differences found between families suggested that while all anthocyanins were dramatically reduced under VHT, coumaroyl and caffeoyl forms were less impacted. These must have been either less degraded, or gene expression may have been deregulated, i.e., downregulation of the biosynthetic genes or upregulation of genes coding for oxidase enzymes, or both. Peroxidase seems to be involved in the degradation of anthocyanins [36,39,40] and has an optimum activity at 40 °C but is completely inactive after 1 h of exposure to temperatures above 50 °C [41], which occurred in VHT treatments. While the activity of enzymes involved in anthocyanin degradation may have been reduced or completely inhibited under VHT, chemical degradation may have occurred instead. Some anthocyanins are known to be chemically more stable than others, due to increased methylation and acylation. In agreement with our observations, several studies found that proportions of methylated anthocyanins including malvidins, which are the most methylated, were increased immediately after heat treatment, as well as acylated forms (except acetyl) at maturity [7,20,30]. In terms of changes in biosynthesis, many genes have been reported to be deregulated with high temperature [9]. In the present study, molecular biology analyses were not performed but if genes were up- or downregulated or enzyme activities modulated, it did not seem to have created any differences under HT treatments and enzymes were probably denatured under extreme heat stress (VHT).

**Figure A2:**
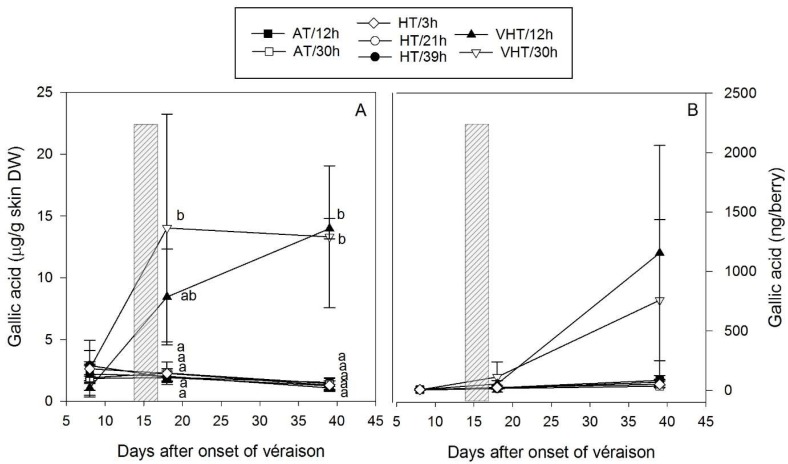
Effect of treatments (temperature intensity: AT, ambient temperature; HT, high temperature; VHT, very high temperature and duration of exposure: varying between 3 and 39 h) on gallic acid in skin (mean ± SD, *n* = 4, except for VHT/12h (*n* = 3) and HT/21h (*n* = 9)) expressed on a skin dry weight basis (**A**) and per berry (**B**). The treatment period (15–17 days after the onset of véraison) is indicated with a grey rectangle. Significant differences between treatments were tested by analysis of variance followed by Tukey test (honestly significant difference) for each sampling date and are indicated with lower case letters (*p* < 0.05).

The decrease in total tannins and free flavan-3-ols clearly correlated with extreme high temperatures, and as for the other flavonoids, some were less reduced than others. Both proportions of galloylated free and polymerised flavan-3-ols were found to be increased under VHT at maturity, concomitant with an increase in skin gallic acid (Figure A2). Very little literature is available apart from another study on Shiraz [12], and grape products processed under extreme heat (>100 °C) [42,43]. *In vivo*, green berries exposed to extreme temperature at the early developmental stage with maximum of 45 °C during the day, also showed an increase in skin galloylation at one sampling date but no significant changes in gallic acid. *In vitro*, the increase in gallic acid is thought to be related to the cleavage of the gallate group already attached to galloylated flavan-3-ols; however, this seems unlikely in our study as all flavan-3-ol galloylation also increased.

Enzymes involved the galloylation process have not all yet been identified and, while some of the genes coding for glycosyltransferases seem to be upregulated under colder conditions in green berries [44], it is possible that other genes involved in the galloylation pathway be upregulated by high temperature. Besides, galloylation may have occurred on polymerised flavan-3-ols as the percentage of galloylation increased despite a smaller tannin size. Interestingly, free flavan-3-ols and tannins were also sometimes impacted differently by extreme high temperature in regards to the proportion of dihydroxylated forms. Dihydroxylated flavan-3-ol monomers were in higher proportions while polymerised dihydroxylated flavan-3-ol subunits were in lower proportions in VHT skins. This could be due to an increase in free flavan-3-ol biosynthesis where dihydroxylated forms were favoured (as observed with flavonols above) as those compounds are still slightly produced after véraison, while tannin polymerisation seem to stop at véraison [45]. 

In addition, in VHT treatments, the number of subunits per polymer was reduced at DAV+39 (mDP_VHT_ = 11.4 ± 0.5), while for the other treatments, polymer size was stable throughout berry ripening and averaged 17.4 ± 0.8 at DAV+39. In vivo, polymer hydrolysis due to heat stress seems unlikely as condensed tannins are very stable polymers and extreme conditions, not only including temperatures above 50 °C but also very low pH, are needed for their depolymerisation [46]. More likely, due to tannin intracellular oxidation, the measured mDP may become lower as the conversion yield decreased with ripening (89% at DAV+8, 75% at DAV+18, and 52% at DAV+39) and became impossible to measure for VHT treatments due to the very low concentration [47]. 

Overall, at maturity, dihydroxylated flavonols and flavan-3-ols were favoured over trihydroxylated forms under VHT, suggesting a modulation of the expression of F3′5′H and F3′H as found in the literature [48]. Indeed, quercetins and catechins were initially the most abundant, and if degraded, could have been replaced quickly through a high biosynthetic turnover. Although di- and trihydroxylated anthocyanin concentrations were both significantly reduced under VHT, their proportions remained the same as in the other treatments (AT and HT) and malvidins (which are trihydroxylated) were the most abundant anthocyanins spanning 52%–67% at maturity regardless of the treatment.

Surprisingly, HT had no effect on concentrations and proportions which contradicts some of the literature, at least for anthocyanins, as they have been found impacted at the bunch level by temperatures lower than those tested in this study. For example, Cabernet Sauvignon bunches exposed to high temperatures before and after véraison (maximum of 37 and 39 °C, respectively) but for a duration of 14 days, exhibited lower concentration of anthocyanins at harvest [7]. In the present study, up to three days of relatively mild heat stress was probably not long enough to impact well-irrigated Shiraz grapevines, pre-grown for two years in an environment where temperatures higher than 40 °C are frequent. This has been previously observed in Australia where flavonoid concentrations were unchanged at harvest in Shiraz whole vines heated for five days at véraison and pre-harvest with maxima over 40 °C [49].

### 2.6. Temperature Effect on Seed Tannins

**Figure 6:**
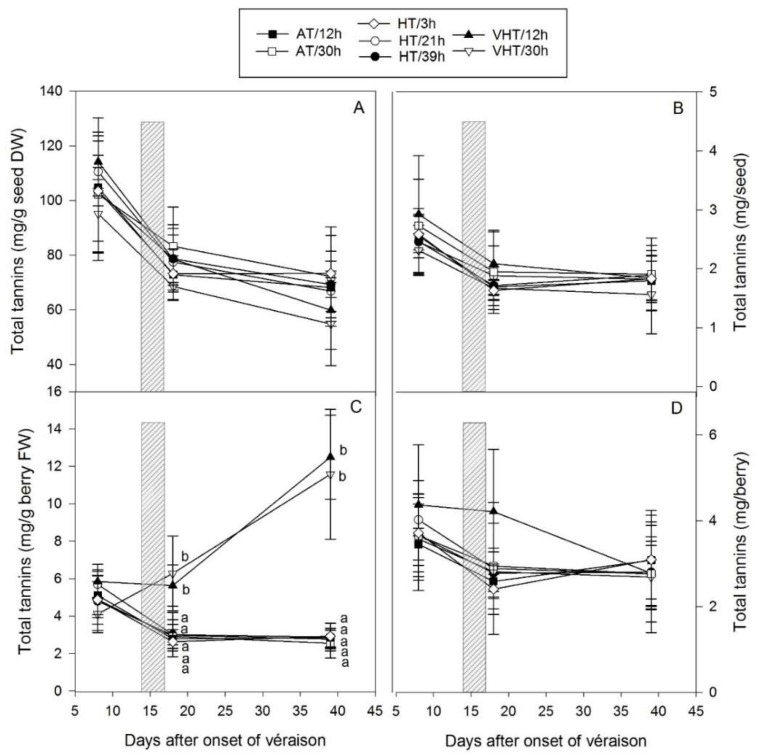
Effect of treatments (temperature intensity: AT, ambient temperature; HT, high temperature; VHT, very high temperature and duration of exposure: varying between 3 and 39 h) on total seed tannins (mean ± SD, *n* = 4, except for VHT/12h (*n* = 3) and HT/21h (*n* = 9)) expressed on seed dry weight (DW) basis (**A**), per seed (**B**), on a berry fresh weight (FW) basis (**C**), and per berry (**D**). The treatment period (15–17 days after the onset of véraison) is indicated with a grey rectangle. Significant differences between treatments were tested by analysis of variance followed by Tukey test (honestly significant difference) for each sampling date and are indicated with lower case letters (*p* < 0.05).

Seed tannins were mostly unaffected by temperature regardless of the intensity and the duration of exposure (Figure 6). In seeds, total tannins slightly decreased from véraison until maturity from 105 to 67 mg/g seed DW (Figure 6A) and from 2.6 to 1.8 mg per seed (Figure 6B). None of these parameters, as well as total concentrations expressed on a FW basis (data no shown), were impacted by temperature even though the seeds of berries under VHT were drier than others (Figure 2D). On a mg/g berry FW basis (Figure 6C), differences in total tannins were present from DAV+18 onwards as berries under VHT were desiccated, and resulting in higher concentrations (12.0 versus 2.8 mg/g), however, this was not reflected in total tannin content where the total averaged 2.8 mg per berry at maturity.

**Figure A3:**
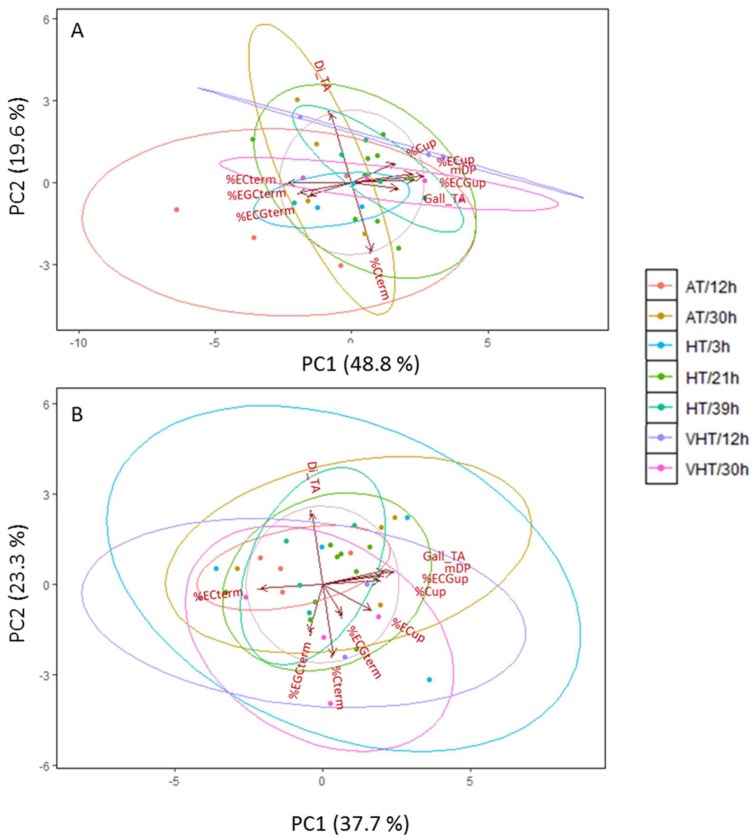
Principal component analysis (PCA) showing the impact of treatments (temperature intensity: AT, ambient temperature; HT, high temperature; VHT, very high temperature and duration of exposure: varying between 3 and 39 h) on the proportion of berry seed tannins just after treatment application (**A**) and at maturity (**B**) with dihydroxylated tannins (Di_TA), mean degree of polymerisation (mDP), percentage of galloylation (Gall_TA) and subunit proportions: C, (+)-Catechin; EC, (−)-Epicatechin; ECG, (−)-Epicatechin gallate; EGC, (−)-Epigallocatechin; term, terminal subunit; up, upper subunits.

No effects on seed tannins, apart from the one driven by berry size/moisture, were observed, suggesting that seeds were protected inside the berry from potential oxidation mechanisms that could happen upon VHT or other abiotic stresses. Seed tannins have been found affected when an intense heat stress was applied after flowering and when seed physiology was largely disrupted [12,50]. However, after véraison, changes in biosynthesis are less likely as seeds reach a maturation phase [19] and genes, after a spike in expression at véraison, become rapidly unexpressed 2 to 4 weeks after véraison [45]. Two PCAs were conducted on the proportions of flavan-3-ol subunits that could be quantified in seeds and more than 60% of variation could be explained by the PC1-2 biplots (Figure A3). Immediately after treatment application, apart from VHT/12h separated from AT/12h, HT/3h and VHT/30h, no clear separation between the other treatments and between temperature intensities were found (Figure A3A). At maturity, even though small changes in proportions seemed to have spread replicates within treatments, none of the treatments were separated from each other and all ellipses were superimposed (Figure A3B). Indeed, no compositional changes were found and none of the following parameters (mDP, %gall) were affected by temperature. In general, a similar inertia has been observed at véraison or harvest for seeds exposed to temperature, light and water stress [35,51,52]. These findings could highlight the economic potential of seeds as tannin content and composition were not affected. Where the damage is too severe and berries are too dry for winemaking, seeds may still be used as by-products with potential further winery [53,54] or health applications [55].

## 3. Materials and Methods 

### 3.1. Plant Materials

Four dormant 7 year-old, own-rooted Shiraz grapevines (*V. vinifera* L.) in 50 L pots filled with premium organic garden mix were moved to a glasshouse to induce budburst mid-November. These had previously been stored in a cool room to enforce a longer period of dormancy as explained by Gouot, et al. [50]. Vines were thinned to six shoots, trained vertically along bamboo stakes, with a total of 10 bunches per vine. Flowering occurred during the second week of December, and véraison started the third week of January. The glasshouse where the experiment was conducted was located at Charles Sturt University, Wagga Wagga (35° S, 147° E), exposed north-east and built with UV-transparent plexiglas^®^. Air temperature, relative humidity and light were monitored as explained in Gouot, et al. [12] and vapour pressure deficit was calculated as a function of relative humidity and temperature (Supplementary Materials, Table S1). Irrigation was managed to ensure no plant water deficit and vines were grown under standard viticultural practices for nutrition and pest and disease management.

### 3.2. Treatment Application and Design

The onset of véraison, defined as the average date when the first berry of each bunch started to turn red [56], occurred on February 6 and treatments were applied at DAV+15–17. Vines were placed on steel mesh benches, and the treatments were applied to individual bunches using blowers connected via flexible ducting to insulated enclosures containing commercial fan heaters as described in Gouot, et al. [12]. Two systems were used to provide two levels of heating above ambient, while a third set of blowers just provided air at ambient temperature. A non-factorial Doehlert design (Table 1) was applied to study the effect of several combinations of independent variables: treatment duration (X1) and temperature intensity (X2). dT was calculated with the temperature of treated bunches compared to untreated bunches for each vine. Three intensities were tested during the day with ambient (AT), high (HT; +8.4 °C), and very high temperature (VHT; +16.7 °C), and HT and VHT applied by differential heating. The AT treatments ensured that any possible effects of mechanical stress and air flow were accounted for in the experimental design. At night, the system was turned off. The duration of the treatments varied between 3 and 39 h with five levels (3, 12, 21, 30, and 39 h) which were applied over a period of three days at a rate of 13 h/day between 7 a.m. and 8 p.m. A total of seven treatments was generated: AT/12h, AT/30h, HT/3h, HT/21h, HT/39h, VHT/12h, and VHT/30h. Each treatment was applied centred around 1:30 p.m. of the second day (DAV+16). Four bunches, one on each vine, were treated as biological replicates (*n* = 4), and HT/21h was repeated several times as it is common for this type of design to replicate the middle point (*n* = 10). The temperature of the air blown onto each bunch was measured by thermocouples and compared to berry surface temperature, estimated by thermal camera imaging (FLIR One for Android, FLIR systems, Wilsonville, OR, USA) as explained in Gouot, et al. [12].

### 3.3. Grape Sampling

Berries were sampled at three times: The first sampling (10 berries) was made one week prior treatment application (DAV+8), at which time each bunch was thinned to 30 berries to reduce heterogeneity between vines [57]. The second sampling (six berries) occurred after the 3 day treatment period (DAV+18), and the third (remaining 24 berries) at harvest, 25 days after the start of the treatment (DAV+39). After determination of berry fresh weight, samples were snap-frozen in liquid nitrogen and stored at −80 °C until processing. Then, berries were slightly thawed and quickly separated into skin, pulp, and seed on ice. Skins were carefully peeled from the berries with tweezers and blotted dry. Skin and seed fresh mass was determined before snap-freezing, followed by manual fine-grinding with a mortar and pestle under liquid nitrogen, freeze-drying and storage at −80 °C until analysis. Pulp was also ground, frozen, and a powder subsample was used to determine TSS as described in Gouot, et al. [50].

### 3.4. Chemical Analysis

#### 3.4.1. Chemical Reagents

Ultrapure water was generated from a Milli-Q Plus purification system (18.2 MΩ cm, Merck Millipore, Bayswater, VIC, Australia). Methanol (HPLC grade, ≥99.9%), acetone (HPLC grade, 99.8%), formic acid (American Chemical Society reagent, ≥98%), trifluoroacetic acid (ReagentPlus^®^, 99%), phloroglucinol (HPLC grade, ≥99%), l-ascorbic acid (reagent grade, ≥98%), ammonium formate (reagent grade, ≥99.9%), (−)-epicatechin (≥90%), quercetin-3-*O*-glucoside (analytical standard, 98%), corticosterone (≥98.5%), ampicillin trihydrate (analytical standard, ≥98%), l-tyrosine (≥99%), l-phenylalanine (≥99%), glutathione reduced (BioReagent, ≥98.0%), caffeic acid (≥98.0%) and gallic acid (≥97.5%), methyl cellulose (1500 cP), and ammonium sulfate (ReagentPlus^®^, ≥99%) were purchased from Sigma-Aldrich (Castle Hill, NSW, Australia). Hydrochloric acid (Trace SELECT, 34%–37%) was purchased from chem-supply (Port Adelaide, SA, Australia). (+)-Catechin, (−)-epicatechin gallate, (−)-epigallocatechin, malvidin-3-*O*-glucoside, dimer B2, and taxifolin (all ≥99.9%) were obtained from Extrasynthese (Genay, France). 

#### 3.4.2. Extraction and Analysis

Freeze-dried skin samples of 14 mg and seed samples of 10 mg were used for extraction as described in Gouot, et al. [50]. Aliquots of 500 µL were transferred into separate tubes and dried with Genevac (EZ-2 Plus, SP Scientific, Ipswich, UK) for tannin and polyphenol analyses. Skin and seed samples for tannin analysis by phloroglucinolysis as well as skin samples for polyphenol analysis were prepared as previously described in Gouot, et al. [50]. Each biological replicate was analysed in duplicate.

Polyphenols, including detailed tannin composition after chemical depolymerisation, were analysed by LC-QqQ-MS as described in Gouot, et al. [50]. Tannin subunits and free-flavan-3-ols were quantified using (+)-catechin, (−)-epicatechin, (−)-epicatechin gallate, and (−)-epigallocatechin standards [58]. Tannin conversion yields (%) were calculated from total tannin concentration in mass measured after phloroglucinolysis as described in Kennedy, et al. [46] and tannin concentration measured by methyl cellulose assay [59]. Anthocyanins and flavonols were quantified using the Multiple Reaction Monitoring (MRM) mode with malvidin-3-*O*-glucoside and quercetin-3-*O*-glucoside as standards, respectively [25]. Individual stock solutions of polyphenol, amino acid and glutathione standards were prepared in 50% MeOH (*v*/*v*) with 1% formic acid and stored at −80 °C. The data sets were normalised to tissue DW. Parameters used to quantify compounds by both analytical methods are available as supplementary material (Supplementary Materials, Table S2).

### 3.5. Chemometrics and Statistical Analysis

**Figure A4:**
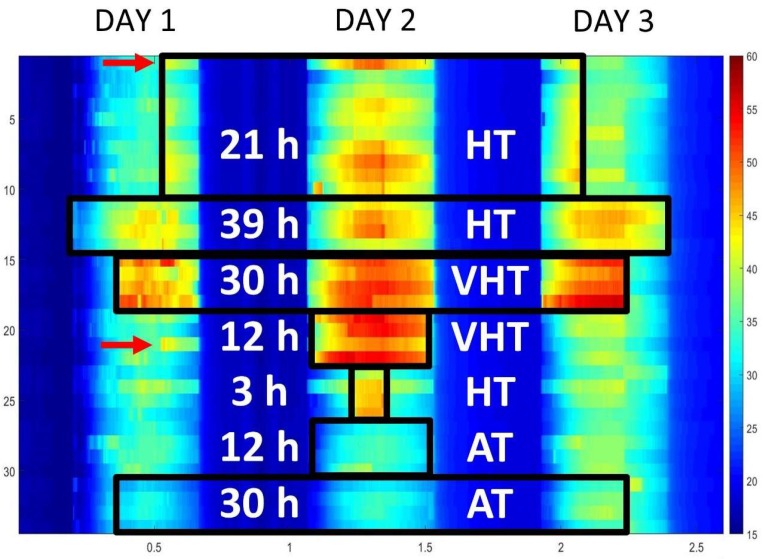
Image display with scaled colours using Matlab of the recorded air temperature during the treatment period of heating treatment (15–17 days after the onset of véraison) for every treated bunches and ordered by treatments (temperature intensity: AT, ambient temperature; HT, high temperature; VHT, very high temperature and duration of exposure: varying between 3 and 39 h). Red arrows point to the two replicates removed from the data set when averages were made.

The average of the four biological replicates was used for graphs and differences between treatments were tested by one-way analysis of variance (ANOVA) followed by Tukey honestly significant difference (HSD)’s test for mean comparison. Two samples were excluded from the data set as the heat treatment applications had clearly failed: one replicate of HT/21h which was excessively heated and damaged and one replicate of VHT/12h which was insufficiently heated (Figure A4). 

A heatmap and clustergram analysis (“heatmap.2” R package) was performed on the average metabolite concentration after normalisation in order to construct separate metabolite correlation matrices for three heat intensities and five durations of exposure, based on the entire set of samples from each treatment. PCAs on the percentage of flavonoid sub-families were separately conducted for each sampling date after treatment application. Both analyses were performed using R (version 3.5.1).

MLRs were completed with Matlab (R2017b) to model each flavonoid sub-family total concentrations in the skin with the coded values of the Doehlert design (dT, duration and interaction between the two). The contribution of each parameter to the prediction was provided by the regression coefficients and the model quality was assessed using the R^2^ values between predicted and experimental values.

## 4. Conclusions

With these new results, the mechanistic response of anthocyanins and flavonols to high temperature is becoming clearer. Flavonols, known as UV-protectors in grapes, seem indirectly affected by high temperature while being degraded under extreme high temperature. The sole effect of high temperature, without excessive light stimuli and outside of the main biosynthesis period (véraison), had no impact on anthocyanins although they were dramatically reduced in damaged berries. However, for tannins and flavan-3-ols, a lack of knowledge in biological and chemical processes involved in synthesis and degradation means that hypotheses made above should be tested in vitro or using solid-state chemistry. The present experiment demonstrated that, in the domain of study, maximum temperature was more critical than duration of exposure. Shiraz berries exposed to maxima around 46 °C showed little physiological differences compared to ambient temperature treatments, and flavonoids were also unaffected by high temperature which is not uncommon in the context of Australian summer weather. However, maxima of 54 °C led to total berry desiccation with complete metabolism shut-down and apparent rapid cell death, suggesting that the threshold for damage was around 50–53 °C for well-irrigated Shiraz mid-ripening. Hence, if heatwave maxima above 40 °C become increasingly frequent, the provision of adequate shade through foliage cover, shade cloth or sunscreen will be needed to maintain fruit surface temperature as low as possible. Under the experimental conditions used in this study, VHT berry final composition was the same regardless of the duration. In the vineyard, if berries exhibit the same damage as observed in this study after an intense heatwave, one way to try and minimise the extent of subsequent yield loss could be to harvest earlier before berries are completely desiccated. However, this practice needs to be tested under real vineyard conditions with measure of the possible colour alteration and mouthfeel and volatile profile modifications.

## Figures and Tables

**Table 1:** Doehlert design used to test two experimental parameters related to heatwaves: X1, duration of exposure with five levels (ranging from 3 to 39 h) and X2, temperature intensity with three levels (AT: ambient temperature; HT: high temperature; VHT: very high temperature). Air was blown onto all treated bunches and average (T_mean_) and maximum (T_max_) were recorded while differential temperatures (dT) were calculated by comparison with untreated bunches (*n* = 4, except for VHT/12h (*n* = 3) and HT/21h (*n* = 9)).

Treatments	Coded Values		Targeted Values ^1^		Experimental T Values		
	*X_1_*	*X_2_*	X1 (h)	X2 (°C)	dT (°C)	T_mean_ (°C)	T_max_ (°C)
AT/12h	−0.5	−0.866	12	0.01	+0.05	31.2	35.9
AT/30h	0.5	−0.866	30	0.01	−1.8	31.6	33.3
HT/3h	−1	0	3	+8.36	+10.2	43.8	45.7
HT/21h	0	0	21	+8.36	+7.3	37.6	45.1
HT/39h	1	0	39	+8.36	+9.0	39.7	46.7
VHT/12h	−0.5	0.866	12	+16.71	+19.8	49.9	55.1
VHT/30h	0.5	0.866	30	+16.71	+14.6	46.6	52.2

^1^ Targeted values were calculated from the coded values using the formula: Xi = mean + *X_i_* × step, where for duration (*X_1_*): mean = 21 h and step = 18 h, and for temperature (*X_2_*): mean = 8.36 °C and step = 9.64 °C

**Table 2:** Model coefficients (b_i_) obtained after multiple linear regression (MLR) analysis using the experimental coded Doehlert design parameters, duration and temperature differential (dT), for each total flavonoid concentration (mg/g skin dry weight) at two sampling dates: 18 days after onset of véraison (DAV+18) and at maturity (DAV+39), and *, **, and *** indicates significant effect at *p* < 0.05, 0.01, and 0.001 respectively.

Coefficients		Anthocyanins		Flavonols		Flavan-3-ols		Tannins	
		DAV+18	DAV+39	DAV+18	DAV+39	DAV+18	DAV+39	DAV+18	DAV+ 39
Constant	b_0_	12.896 ***	12.828 ***	5.313 ***	5.436 ***	0.604 ***	0.171 ***	82.069 ***	37.807 ***
Duration	b_1_	0.724	−0.782	0.512	0.320	0.0144	−0.0129	−2.495	−0.601
dT	b_2_	−7.070 ***	−8.373 ***	−2.070 *	−2.667 **	−0.3 ***	0.104 ***	−35.867 ***	−21.593 ***
Duration * dT	b_12_	−5.233 *	−2.366	−1.685	−0.822	−0.0733	−0.0207	−4.603	−0.472
Regression	R^2^	0.426	0.597	0.193	0.246	0.401	0.531	0.389	0.568

## Supplementary Materials

Supplementary materials are available.

## References

[1] Perkins S.E., Alexander L.V. (2013). On the measurement of heat waves. J. Clim..

[2] Perkins-Kirkpatrick S., White C., Alexander L., Argüeso D., Boschat G., Cowan T., Evans J., Ekström M., Oliver E., Phatak A. (2016). Natural hazards in Australia: Heatwaves. Clim. Chang..

[3] Perkins-Kirkpatrick S., Pitman A. (2018). Extreme events in the context of climate change. Public Health Res. Pract..

[4] Webb L., Whiting J., Watt A., Hill T., Wigg F., Dunn G., Needs S., Barlow E.W.R. (2010). Managing Grapevines through Severe Heat: A Survey of Growers after the 2009 Summer Heatwave in South-eastern Australia. J. Wine Res..

[5] Frioni T., Tombesi S., Luciani E., Sabbatini P., Berrios J.G., Palliotti A. (2019). Kaolin treatments on Pinot noir grapevines for the control of heat stress damages. BIO Web Conf..

[6] Sweetman C., Sadras V.O., Hancock R.D., Soole K.L., Ford C.M. (2014). Metabolic effects of elevated temperature on organic acid degradation in ripening *Vitis vinifera* fruit. J. Exp. Bot..

[7] Lecourieux F., Kappel C., Pieri P., Charon J., Pillet J., Hilbert G., Renaud C., Gomès E., Delrot S., Lecourieux D. (2017). Dissecting the biochemical and transcriptomic effects of a locally applied heat treatment on developing Cabernet Sauvignon grape berries. Front. Plant. Sci..

[8] Drappier J., Thibon C., Rabot A., Geny-Denis L. (2017). Relationship between wine composition and temperature: Impact on Bordeaux wine typicity in the context of global warming—Review. Crit. Rev. Food Sci. Nutr..

[9] Gouot J.C., Smith J.P., Holzapfel B.P., Walker A.R., Barril C. (2019). Grape berry flavonoids: A review of their biochemical responses to high and extreme high temperatures. J. Exp. Bot..

[10] Doehlert D.H. (1970). Uniform shell designs. Appl. Stat..

[11] Ferreira S.L.C., dos Santos W.N.L., Quintella C.M., Neto B.B., Bosque-Sendra J.M. (2004). Doehlert matrix: A chemometric tool for analytical chemistry—review. Talanta.

[12] Gouot J.C., Smith J.P., Holzapfel B.P., Barril C. (2019). Impact of short temperature exposure of *Vitis vinifera* L. cv. Shiraz grapevine bunches on berry development, primary metabolism and tannin accumulation. Environ. Exp. Bot..

[13] Kliewer W. (1977). Effect of high temperatures during the bloom-set period on fruit-set, ovule fertility, and berry growth of several grape cultivars. Am. J. Enol. Viticult..

[14] Reshef N., Fait A., Agam N. (2019). Grape berry position affects the diurnal dynamics of its metabolic profile. Plant. Cell Environ..

[15] Reshef N., Walbaum N., Agam N., Fait A. (2017). Sunlight Modulates Fruit Metabolic Profile and Shapes the Spatial Pattern of Compound Accumulation within the Grape Cluster. Front. Plant Sci..

[16] Keller M. (2015). The Science of Grapevines: Anatomy and Physiology.

[17] Perkins S. Scorcher. http://scorcher.org.au.

[18] Schrader L.E., Zhang J., Duplaga W.K. (2001). Two types of sunburn in apple caused by high fruit surface (peel) temperature. Plant. Health Prog..

[19] Rousserie P., Rabot A., Geny-Denis L. (2019). From flavanols biosynthesis to wine tannins: What place for grape seeds?. J. Agric. Food Chem..

[20] Tarara J.M., Lee J., Spayd S.E., Scagel C.F. (2008). Berry Temperature and Solar Radiation Alter Acylation, Proportion, and Concentration of Anthocyanin in Merlot Grapes. Am. J. Enol. Viticult..

[21] Azuma A., Yakushiji H., Koshita Y., Kobayashi S. (2012). Flavonoid biosynthesis-related genes in grape skin are differentially regulated by temperature and light conditions. Planta.

[22] Rentzsch M., Schwarz M., Winterhalter P. (2007). Pyranoanthocyanins–an overview on structures, occurrence, and pathways of formation. Trends Food Sci. Tech..

[23] Fulcrand H., Benabdeljalil C., Rigaud J., Cheynier V., Moutounet M. (1998). A new class of wine pigments generated by reaction between pyruvic acid and grape anthocyanins. Phytochemistry.

[24] Bakker J., Timberlake C.F. (1997). Isolation, identification, and characterization of new color-stable anthocyanins occurring in some red wines. J. Agric. Food Chem..

[25] Pinasseau L., Vallverdú-Queralt A., Verbaere A., Roques M., Meudec E., Le Cunff L., Peros J.-P., Ageorges A., Sommerer N., Boulet J.-C. (2017). Cultivar diversity of grape skin polyphenol composition and changes in response to drought investigated by LC-MS based metabolomics. Front. Plant. Sci..

[26] Arapitsas P., Oliveira J., Mattivi F. (2015). Do white grapes really exist?. Food Res. Int..

[27] Zhao Q., Duan C.-Q., Wang J. (2010). Anthocyanins profile of grape berries of *Vitis amurensis*, its hybrids and their wines. Int. J. Mol. Sci..

[28] Degu A., Ayenew B., Cramer G.R., Fait A. (2016). Polyphenolic responses of grapevine berries to light, temperature, oxidative stress, abscisic acid and jasmonic acid show specific developmental-dependent degrees of metabolic resilience to perturbation. Food Chem..

[29] Rustioni L. (2017). Oxidized polymeric phenolics: Could they be considered photoprotectors?. J. Agric. Food Chem..

[30] Mori K., Sugaya S., Gemma H. (2005). Decreased anthocyanin biosynthesis in grape berries grown under elevated night temperature condition. Sci. Hortic..

[31] Spayd S.E., Tarara J.M., Mee D.L., Ferguson J.C. (2002). Separation of sunlight and temperature effects on the composition of *Vitis vinifera* cv. Merlot berries. Am. J. Enol. Viticult..

[32] Downey M., Dokoozlian N.K., Krstic M.P. (2006). Cultural practice and environmental impacts on the flavonoid composition of grapes and wine: A review of recent research. Am. J. Enol. Viticult..

[33] Wahid A., Gelani S., Ashraf M., Foolad M.R. (2007). Heat tolerance in plants: An overview. Environ. Exp. Bot..

[34] Downey M.O., Harvey J.S., Robinson S.P. (2003). Synthesis of flavonols and expression of flavonol synthase genes in the developing grape berries of Shiraz and Chardonnay (Vitis vinifera L.). Aust. J. Grape Wine Res..

[35] Cohen S.D., Tarara J.M., Kennedy J.A. (2008). Assessing the impact of temperature on grape phenolic metabolism. Anal. Chim. Acta..

[36] Pastore C., Dal Santo S., Zenoni S., Movahed N., Allegro G., Valentini G., Filippetti I., Tornielli G.B. (2017). Whole plant temperature manipulation affects flavonoid metabolism and the transcriptome of grapevine berries. Front. Plant Sci..

[37] Gaiotti F., Pastore C., Filippetti I., Lovat L., Belfiore N., Tomasi D. (2018). Low night temperature at veraison enhances the accumulation of anthocyanins in Corvina grapes (*Vitis Vinifera* L.). Sci. Rep..

[38] Gao-Takai M., Katayama-Ikegami A., Matsuda K., Shindo H., Uemae S., Oyaizu M. (2019). A low temperature promotes anthocyanin biosynthesis but does not accelerate endogenous abscisic acid accumulation in red-skinned grapes. Plant. Sci..

[39] Mori K., Goto-Yamamoto N., Kitayama M., Hashizume K. (2007). Loss of anthocyanins in red-wine grape under high temperature. J. Exp. Bot..

[40] Movahed N., Pastore C., Cellini A., Allegro G., Valentini G., Zenoni S., Cavallini E., D’Incà E., Tornielli G.B., Filippetti I. (2016). The grapevine VviPrx31 peroxidase as a candidate gene involved in anthocyanin degradation in ripening berries under high temperature. J. Plant. Res..

[41] Saeidian S., Ghasemifar E. (2013). Effect of Temperature on Guaiacol Peroxidase of Pyrus communis. Int. Lett. Nat. Sci..

[42] Chamorro S., Goñi I., Viveros A., Hervert-Hernández D., Brenes A. (2012). Changes in polyphenolic content and antioxidant activity after thermal treatments of grape seed extract and grape pomace. Eur. Food Res.Technol..

[43] Ross C.F., Hoye C., Fernandez-Plotka V.C. (2011). Influence of heating on the polyphenolic content and antioxidant activity of grape seed flour. J. Food Sci..

[44] Rienth M., Torregrosa L., Sarah G., Ardisson M., Brillouet J.-M., Romieu C. (2016). Temperature desynchronizes sugar and organic acid metabolism in ripening grapevine fruits and remodels their transcriptome. BMC Plant. Biol..

[45] Bogs J., Downey M.O., Harvey J.S., Ashton A.R., Tanner G.J., Robinson S.P. (2005). Proanthocyanidin synthesis and expression of genes encoding leucoanthocyanidin reductase and anthocyanidin reductase in developing grape berries and grapevine leaves. Plant. Physiol..

[46] Kennedy J.A., Jones G.P. (2001). Analysis of proanthocyanidin cleavage products following acid-catalysis in the presence of excess phloroglucinol. J. Agric. Food Chem..

[47] Brillouet J.-M., Fulcrand H., Carrillo S., Rouméas L., Romieu C. (2017). Isolation of Native Proanthocyanidins from Grapevine (*Vitis vinifera*) and Other Fruits in Aqueous Buffer. J. Agric. Food Chem..

[48] Mori K., Goto-Yamamoto N., Kitayama M., Hashizume K. (2007). Effect of high temperature on anthocyanin composition and transcription of flavonoid hydroxylase genes in ‘Pinot noir’grapes (*Vitis vinifera*). J. Hortic. Sci. Bio. Tech..

[49] Sommer K., Edwards E., Unwin D., Mazza M., Downey M. (2012). Strategies to Maintain Productivity and Quality in a Changing Environment-Impacts of Global Warming on Grape and Wine Production.

[50] Gouot J.C., Smith J.P., Holzapfel B.P., Barril C. (2019). Single and cumulative effects of whole-vine heat events on Shiraz berry composition. OENO One.

[51] Downey M., Harvey J.S., Robinson S.P. (2004). The effect of bunch shading on berry development and flavonoid accumulation in Shiraz grapes. Aust. J. Grape Wine Res..

[52] Roby G., Harbertson J.F., Adams D.A., Matthews M.A. (2004). Berry size and vine water deficits as factors in winegrape composition: Anthocyanins and tannins. Aust. J. Grape Wine Res..

[53] García-Marino M., Rivas-Gonzalo J.C., Ibáñez E., García-Moreno C. (2006). Recovery of catechins and proanthocyanidins from winery by-products using subcritical water extraction. Anal. Chim. Acta..

[54] Romanini E., McRae J.M., Colangelo D., Lambri M. (2019). First trials to assess the feasibility of grape seed powder (GSP) as a novel and sustainable bentonite alternative. Food Chem..

[55] Shrikhande A.J. (2000). Wine by-products with health benefits. Food Res. Int..

[56] Coombe B. (1995). Growth stages of the grapevine: Adoption of a system for identifying grapevine growth stages. Aust. J. Grape Wine Res..

[57] Gouthu S., O’Neil S.T., Di Y., Ansarolia M., Megraw M., Deluc L.G. (2014). A comparative study of ripening among berries of the grape cluster reveals an altered transcriptional programme and enhanced ripening rate in delayed berries. J. Exp. Bot..

[58] Pinasseau L., Verbaere A., Roques M., Meudec E., Vallverdú-Queralt A., Terrier N., Boulet J.-C., Cheynier V., Sommerer N. (2016). A fast and robust UHPLC-MRM-MS method to characterize and quantify grape skin tannins after chemical depolymerization. Molecules.

[59] Sarneckis C.J., Dambergs R., Jones P., Mercurio M., Herderich M.J., Smith P. (2006). Quantification of condensed tannins by precipitation with methyl cellulose: Development and validation of an optimised tool for grape and wine analysis. Aust. J. Grape Wine Res..

