# Supplementary material for: Grape Berry Flavonoid Responses to High Bunch Temperatures Post Véraison: Effect of Intensity and Duration of Exposure
Source: Molecules. 2019 Nov 27;24(23):4341. doi: 10.3390/molecules24234341 (PMC6930521; doi:10.3390/molecules24234341)
Supplement: Supplementary file 1 [file molecules-24-04341-s001.zip › Supplementary files/Table S1.docx]

**Table S1.** Mean, maximum (max) and minimum (min) temperature (T), relative humidity (RH) and vapour pressure deficit (VPD) recorded during the treatment application for all treatments (temperature intensity: Amb, ambient temperature; +8.4, high temperature; +16.7, very high temperature and duration of exposure: varying between 3 and 39 h).

| Period | Treatment | Rate of treatment application | Tmean (°C) | Tmax (°C) | Tmin (°C) | RHmax (%) | RHmin (%) | VPDmax (kPa) | VPDmin (kPa) |
| --- | --- | --- | --- | --- | --- | --- | --- | --- | --- |
|  |  |  | (During the treatment application) | | | | | | |
| Day 1 (7AM-8PM) | Reference | - | 28.7 | 36.2 | 16.1 | 54.4 | 25.1 | 4.7 | 0.8 |
|  | +8.4 °C for 21 h | 4 h | 35.9 | 41.5 | 27.9 |  |  | 6.3 | 0.8 |
|  | +8.4 °C for 39 h | 13 h | 36.9 | 44.4 | 22.6 |  |  | 7.8 | 1.8 |
|  | +16.7 °C for 30 h | 8.5 h | 44.6 | 50.9 | 30.7 |  |  | 10.9 | 0.8 |
|  | +16.7 °C for 12 h | Not applied | 30.4 | 38.8 | 16.3 |  |  | 5.3 | 0.9 |
|  | +8.4 °C for 3 h | Not applied | 29.6 | 36.6 | 16.5 |  |  | 5.0 | 0.9 |
|  | Amb for 12 h | Not applied | 29.4 | 37.0 | 15.9 |  |  | 4.7 | 0.8 |
|  | Amb^1^ for 30 h | 8.5 h | 28.9 | 32.1 | 23.3 |  |  | 3.3 | 1.0 |
| Day 2 (7AM-8PM) | Reference | - | 30.8 | 37.0 | 18.1 | 77.2 | 28.8 | 4.6 | 0.5 |
|  | +8.4 °C for 21 h | 13 h | 38.5 | 45.1 | 22.7 |  |  | 7.9 | 1.0 |
|  | +8.4 °C for 39 h | 13 h | 40.8 | 46.7 | 23.7 |  |  | 8.9 | 1.4 |
|  | +16.7 °C for 30 h | 13 h | 46.8 | 52.2 | 24.2 |  |  | 12.0 | 1.5 |
|  | +16.7 °C for 12 h | 12 h | 49.9 | 55.1 | 27.4 |  |  | 13.9 | 0.7 |
|  | +8.4 °C for 3 h | 3 h | 43.8 | 45.7 | 37.0 |  |  | 8.3 | 0.6 |
|  | Amb^1^ for 12 h | 12 h | 31.2 | 35.9 | 20.4 |  |  | 4.3 | 0.5 |
|  | Amb^1^ for 30 h | 13 h | 28.9 | 33.3 | 18.1 |  |  | 3.5 | 0.5 |
| Day 3 (7AM-8PM) | Reference | - | 32.6 | 39.0 | 20.9 | 66.6 | 26.5 | 5.1 | 1.0 |
|  | +8.4 °C for 21 h | 4 h | 36.5 | 43.5 | 26.1 |  |  | 6.8 | 1.7 |
|  | +8.4 °C for 39 h | 13 h | 41.4 | 45.8 | 28.2 |  |  | 8.1 | 2.3 |
|  | +16.7 °C for 30 h | 8.5 h | 48.3 | 52.1 | 29.7 |  |  | 12.4 | 1.9 |
|  | +16.7 °C for 12 h | Not applied | 34.9 | 40.9 | 21.6 |  |  | 6.0 | 1.2 |
|  | +8.4 °C for 3 h | Not applied | 33.3 | 37.9 | 21.3 |  |  | 5.1 | 1.1 |
|  | Amb for 12 h | Not applied | 33.0 | 38.7 | 20.7 |  |  | 5.0 | 1.0 |
|  | Amb^1^ for 30 h | 8.5 h | 32.1 | 35.2 | 21.0 |  |  | 5.2 | 1.0 |

^1^Fan blowing air at ambient temperature
