# Supplementary material for: Grape Berry Flavonoid Responses to High Bunch Temperatures Post Véraison: Effect of Intensity and Duration of Exposure
Source: Molecules. 2019 Nov 27;24(23):4341. doi: 10.3390/molecules24234341 (PMC6930521; doi:10.3390/molecules24234341)
Supplement: Supplementary file 1 [file molecules-24-04341-s001.zip › Supplementary files/Table S2.docx]

|  |  |  |  | | | |  |
| --- | --- | --- | --- | --- | --- | --- | --- |
| **Table S2.** List of quantified compounds, MRM parameters such as ionisation mode (+: positive; -: negative), transition ions used (m/z: mass over charge; P: precursors, Qt: quantifiers, Ql: qualifier), retention time (RT) and calibration range. Compound abbreviations: Ac: acetyl; Cou: coumaroyl; Caff: caffeoyl; Glc: glucoside; Glucu: glucuronide; C: (+)-catechin; EC: (-)-epicatechin; ECG: (-)-epicatechin gallate; GC: (+)-gallocatechin; EGC: (-)-epigallocatechin; Xterm: X terminal subunit; Xup: X upper subunit; AA: amino acids; OT: others; HB: hydroxybenzoic acids; HC: hydroxycinnamic acids. | | | | | | | |
| **Family** | **Compounds** | **Ionisation Mode** | **Ions for MRM** | | | | **Calibration range** |
|  |  |  | **m/z (P)** | **m/z (Qt)** | **m/z (Ql)** | **RT** |  |
| NATIVE ANTHOCYANINS | Delphinidin-3-O-Glc | + | 465.2 | 303.1 | 229.0 | 12.5 | As malvidin-3-O-glucoside eq. (0.01-250 mg/L)  y = -151.67x^2^ + 191995x + 79580  R² = 0.999 |
|  | Cyanidin-3-O-Glc | + | 449.2 | 287.1 | 137.2 | 13.6 |  |
|  | Petunidin-3-O-Glc | + | 479.2 | 317.2 | 302.3 | 14.2 |  |
|  | Peonidin-3-O-Glc | + | 463.2 | 301.2 | 286.1 | 14.8 |  |
|  | Malvidin-3-O-Glc | + | 493.2 | 331.2 | 315.0 | 15.1 |  |
|  | Delphinidin-3-O-AcGlc | + | 507.2 | 303.0 | 229.0 | 16.1 |  |
|  | Cyanidin-3-O-AcGlc | + | 491.2 | 287.1 | 137.0 | 17.2 |  |
|  | Petunidin-3-O-AcGlc | + | 521.2 | 317.2 | 302.3 | 17.6 |  |
|  | Peonidin-3-O-AcGlc | + | 505.2 | 301.1 | 286.2 | 19.2 |  |
|  | Malvidin-3-O-AcGlc | + | 535.2 | 331.2 | 315.0 | 19.5 |  |
|  | Delphinidin-3-O-CouGlc^1^ | + | 611.2 | 303.1 | 229.1 | 18.2; 21.1 |  |
|  | Cyanidin-3-O-CouGlc^1^ | + | 595.2 | 287.2 | 157.1 | 20.1; 23.1 |  |
|  | Petunidin-3-O-CouGlc^1^ | + | 625.2 | 317.2 | 302.3 | 21.0; 23.4 |  |
|  | Peonidin-3-O-CouGlc^1^ | + | 609.2 | 301.2 | 286.2 | 22.9; 24.0 |  |
|  | Malvidin-3-O-CouGlc^1^ | + | 639.2 | 331.2 | 315.2 | 23.0; 24.3 |  |
|  | Pelargonidin-3-O-CouGlc^1^ | + | 579.0 | 271.0 | / | 22.3; 24.0 |  |
|  | Peonidin-3-O-CaffGlc | + | 625.2 | 301.2 | 286.0 | 22.3 |  |
|  | Malvidin-3-O-CaffGlc | + | 655.2 | 331.2 | 315.1 | 22.4 |  |
| PYRANO ANTHO | Pyranopeonidin-3-O-Glc | + | 487.2 | 325.1 | 310.2 | 15.4 |  |
|  | Pyranomalvidin-3-O-Glc | + | 517.2 | 355.1 | 339.2 | 16.0 |  |
|  | Carboxypyranopeonidin-3-O-Glc | + | 531.2 | 369.2 | 297.2 | 15.9 |  |
|  | Carboxypyranomalvidin-3-O-Glc | + | 561.2 | 399.1 | 383.1 | 16.2 |  |
| FLAVONOLS | Myricetin-3-O-Glucu | + | 495.2 | 319.1 | 89.1 | 15.0 | As quercetin-3-O-glucoside eq. (0.02-100 mg/L)  y = -133.62x^2^ + 46653x - 688.08  R² = 1 |
|  | Myricetin-3-O-Glc | + | 481.2 | 319.1 | 123.1 | 15.2 |  |
|  | Quercetin-3-O-Glucu | + | 479.2 | 303.1 | 85.1 | 16.7 |  |
|  | Quercetin-3-O-Glc | + | 465.2 | 303.0 | 153.0 | 16.8 |  |
|  | Laricitin-3-O-Glc | + | 495.2 | 331.1 | 85.0 | 17.1 |  |
|  | Laricitin-3-O-Glucu | + | 509.5 | 331.4 | / | 17.3 |  |
|  | Kaempfero-3-O-Glucu | + | 449 | 287.3 | 153.0 | 19.1 |  |
|  | Kaempferol-3-O-Glc | + | 463.4 | 287.3 | 153.0 | 19.5 |  |
|  | Isorhamnetin-3-O-Glc | + | 479.5 | 317.4 | 153.0 | 19.6 |  |
|  | Syringetin-3-O-Glc | + | 509.1 | 347.0 | 287.0 | 19.9 |  |
|  | Isorhamnetin-3-O-Glucu | + | 493.4 | 317.3 | 153.0 | 20.3 |  |
|  | Syringetin-3-O-Glucu | + | 523.1 | 347.0 | 287.0 | 20.7 |  |
| FLAVAN-3-OLS (free) | GC | + | 307.1 | 139.0 | 151.0 | 2.4 | As EGC eq.  (0.01-100 mg/L)  y = -70.673x^2^ + 48437x + 53642, R² = 0.9995 |
|  | EGC | + | 307.1 | 139.0 | 151.0 | 7.2 |  |
|  | C | + | 291.1 | 139.0 | 123.1 | 7.2 | As C eq. (0.01-100 mg/L)  y = -90.02x^2^ + 40505x + 50991, R² = 0.999 |
|  | EC | + | 291.1 | 139.0 | 123.1 | 11.9 | As EC eq. (0.01-100 mg/L)  y = -33.646x^2^ + 34879x + 100198, R² = 0.999 |
|  | ECG | + | 443.1 | 123.1 | 273.1 | 14.6 | As ECG eq. (0.01-150 mg/L)  y = -26.393x^2^ + 18993x + 24049, R² = 0.9992 |
|  | Dimer B1 | + | 579.2 | 127.0 | 289.0 | 7.2 | As dimer B2 eq.  (0.01-30 mg/L)  y = 10682x + 190.63, R² = 0.9999 |
|  | Dimer B2 | + | 579.2 | 127.0 | 289.0 | 10.4 |  |
| AA | Tyrosine | + | 182.0 | 136.1 | 165.1 | 1.1 | As tyrosine eq. (0.3-20 mg/L)  y = 289042x + 76529, R² = 0.9985 |
|  | Phenylalanine | + | 166.1 | 120.1 | 103.1 | 2.8 | As phenylalanine eq. (0.3-20 mg/L)  y = 900088x + 371425, R² = 0.9978 |
| OT | Glutathione reduced | + | 308.1 | 179.0 | 162.0 | 0.7 | As glutathione reduced eq.  (0.03-80 mg/L)  y = 80668x + 77544, R² = 0.999 |
| HB | Caffeic acid | - | 179.0 | 135.0 | 79.0 | 7.5 | As caffeic acid eq. (0.1-20 mg/L)  y = -663.17x^2^ + 43396x + 2146, R² = 0.9998 |
|  | Coutaric acid^2^ | - | 295.1 | 163.1 | 119.1 | 7.3 |  |
|  | Fertaric acid | - | 325.0 | 193.0 | 149.0 | 9.9 |  |
|  | Caftaric acid^2^ | - | 311.0 | 179.0 | 149.0 | 4.7 |  |
| HC | Gallic acid | - | 169.0 | 125.0 | 79.0 | 1.1 | As gallic acid eq. (0.5-100 mg/L)  y = -58.333x^2^ + 13907x + 1542.5, R² = 0.9999 |
|  | Glucogallin | - | 331.0 | 169.0 | 151 | 1.1 |  |
| PROANTHOCYANIDINS^3^ | EGCup | + | 431.2 | 127.1 | 289.1 | 0.7 | As EC eq.^4^  (0.01-350 µmol/L)  y = -10.485x^2^ + 11791x + 1391 R² = 1 |
|  | ECGup | + | 567.2 | 247.2 | 153.1 | 3.9 |  |
|  | Cup | + | 415.1 | 127.1 | 289.1 | 1.1 |  |
|  | ECup | + | 415.1 | 127.1 | 289.1 | 1.3 |  |
|  | Cterm | + | 291.1 | 139.0 | 123.1 | 2.9 | As C eq. (0.01-170 µmol/L)  y = -21.657x^2^ + 17201x + 89.378 R² = 1 |
|  | EGCterm | + | 307.1 | 139.0 | 151.0 | 2.8 | As EGC eq. (0.01-170 µmol/L)  y = -18.776x2 + 20457x - 1803.3 R² = 1 |
|  | GCterm | + | 307.1 | 139.0 | 151.0 | 1.0 |  |
|  | ECterm | + | 291.1 | 139.0 | 123.1 | 5.9 | As EC eq. |
|  | ECGterm | + | 443.1 | 123.1 | 273.1 | 6.9 | As ECG eq. (0.01-170 µmol/L)  y = -10.636x2 + 7477.3x + 189.96 R² = 1 |
| ^1^ cis and trans isomers  ^2^ co-eluted isomers  ^3^ tannin composition after cleavage of the polymer by phloroglucinolysis – different LC method to other polyphenols.  ^4^ concentrations calculated with correction values established between mass spectrometry and UV detectors | | | | | | | |
